# Risk factors and predictive modeling of postpartum depression among postpartum women: empirical evidence from Chongqing, China

**DOI:** 10.3389/fpubh.2026.1725970

**Published:** 2026-02-13

**Authors:** Jiaming Jiang, Guichuan Lai, Wenlong Li, Yingcheng Liu, Haijiao Zeng, Tian Liu, Xinjing Liu, Qian Wang, Biao Xie, Xiaoni Zhong

**Affiliations:** 1 Department of Health Statistics, School of Public Health, Chongqing Medical University, Chongqing, China; 2 Research Center for Medicine and Social Development, Chongqing Medical University, Chongqing, China

**Keywords:** China, logistic regression, postpartum depression, predictive model, risk factors

## Abstract

**Background:**

Postpartum depression (PPD) is a prevalent mental health condition that significantly impacts the wellbeing of mothers and their families. Early identification of high-risk women continues to be a challenge in public health practice. This study focuses on postpartum women in Chongqing, China, aiming to identify key psychosocial and demographic risk factors for PPD. Using logistic regression, we constructed and validated a predictive model for early screening, which could guide targeted preventive interventions.

**Methods:**

This cross-sectional study was conducted from January 2018 to July 2019 at four hospitals in Chongqing, China. A total of 892 valid questionnaires were collected based on predefined inclusion and exclusion criteria. Univariate and multivariable logistic regression analyses were performed to identify predictors of PPD and to construct a predictive model. The model’s performance was evaluated in terms of discrimination, calibration, and clinical utility using the area under the receiver operating characteristic curve (AUC), calibration curves, and decision curve analysis (DCA), respectively. The dataset was randomly divided into a training set (70%) for model development and a validation set (30%) for internal validation.

**Results:**

Among the 892 participants, the prevalence of PPD was 10.2%. Multivariable logistic regression analysis identified four independent predictors: low delivery-related knowledge (OR = 5.47, 95% CI: 2.08–14.40), family dysfunction (moderate: 7.03, 3.79–13.02; severe: 5.14, 2.08–12.72), low social support (3.92, 1.06–14.42), and cesarean section (2.31, 1.31–4.09). The AUC of the model was 0.83 in both the training and validation sets. The calibration curve demonstrated good agreement between predicted and observed outcomes, and DCA confirmed its potential clinical utility.

**Conclusion:**

Key risk factors for PPD in this study include low delivery-related knowledge, family dysfunction, low social support, and cesarean section. The developed model performs well in the early identification of high-risk women, enabling timely interventions to improve maternal mental health.

## 1 Introduction

Postpartum depression (PPD) is a prevalent mental health disorder affecting women worldwide, with significant implications for both maternal wellbeing and child development (1). Women experiencing PPD often exhibit symptoms such as persistent sadness, fatigue, feelings of worthlessness, reduced interest in their infants, loss of appetite, and sleep disturbances. These symptoms not only hinder a mother’s ability to provide adequate care and affection but also have enduring negative effects on infant development and family dynamics (2). Epidemiological studies show that the global prevalence of PPD ranges from 5.0% to 26.3% (3), with a reported prevalence of 14.8% in China (4).

The risk factors for PPD are multifactorial, encompassing biological, psychosocial, and sociodemographic domains (5). Biologically, genetic predispositions and postpartum hormonal changes, including disruptions of the hypothalamic–pituitary–adrenal axis and inflammatory responses, are associated with an increased risk of PPD (6–8). Psychosocial factors such as depression, life stressors, inadequate partner support, and poor family relationships are also critical predictors of PPD (9). The quality of family and social support plays a key role in mitigating this risk: strong emotional and instrumental support from family members, particularly the partner, can buffer the effects of stress and reduce the likelihood of PPD. External social support from friends, community resources, and healthcare professionals provides protective benefits by helping mothers navigate postpartum challenges and reducing feelings of isolation (10). In addition, sociodemographic factors such as age, education level, and socioeconomic status further influence the likelihood of developing PPD (11–14).

PPD has been increasingly studied, yet many existing models do not fully capture the multifactorial nature of its risk factors—particularly the combined effects of psychosocial, obstetric, and sociodemographic variables. This gap is especially evident in populations with distinct cultural and healthcare contexts, such as China. Although logistic regression is widely used in PPD prediction because of its interpretability and has shown performance comparable to more complex approaches such as neural networks (15), there remains a need for models that holistically address population-specific determinants. In this study, we used logistic regression to develop a prediction model that integrates these intertwined factors among women in Chongqing, China.

To address these gaps, the present study aimed to identify the major risk factors for PPD among women in Chongqing, China, with a particular focus on psychosocial and sociodemographic dimensions. Using logistic regression, we developed and validated a predictive model for PPD. In this approach, logistic regression estimates the probability of PPD as a weighted combination of risk factors, with each predictor assigned a regression coefficient that quantifies its independent contribution to PPD risk. These coefficients are then transformed into odds ratios, which provide a clear interpretation of how individual factors—such as low social support, family dysfunction, or cesarean section—affect the likelihood of developing PPD. Furthermore, the model is visualized as a nomogram, enabling healthcare providers to calculate individualized risk scores and apply them in clinical or community settings.

The goal of this study is to provide an effective early-warning tool for public health practitioners to identify high-risk individuals and reduce the adverse effects of PPD on mothers and their families. The findings of this study may help inform targeted interventions and improve maternal mental health outcomes in China and globally.

## 2 Methods

### 2.1 Study design

This study adopted a cross-sectional design. Data were collected from January 2018 to July 2019 among postpartum women who attended routine postnatal visits at four hospitals in Chongqing, China. Eligible participants completed self-administered questionnaires that captured pregnancy-related environmental factors as well as postpartum depressive symptoms. Based on predefined inclusion and exclusion criteria, a total of 892 valid questionnaires were included in the final analysis.

### 2.2 Participant selection

This study used data from a project funded by the National Natural Science Foundation of China (Grant No. 71573027). The project used a stratified sampling method based on regional economic levels within Chongqing Municipality in Southwest China. Four healthcare institutions were selected as study sites: North Chongqing Maternal and Child Health Hospital in Yubei District, Jiangjin District Maternal and Child Health Hospital, Yunyang County Maternal and Child Health Hospital, and Dianjiang County People’s Hospital. Data were collected in the postpartum wards and outpatient clinics of these hospitals.

A structured, self-administered paper-based questionnaire was used to collect data through face-to-face interviews. The instrument consisted of the Chinese version of the Self-Rating Depression Scale (SDS) and additional items on sociodemographic, psychosocial, and obstetric characteristics. The questionnaire was developed based on an extensive review of domestic and international literature. To ensure its applicability and scientific rigor to the Chinese population, a preliminary version was piloted in a sample of postpartum women. The content was then repeatedly discussed and refined in consultation with a multidisciplinary panel of experts in epidemiology, health statistics, psychology, and obstetrics before being finalized. Data were collected from ward nurses who had received standardized training in survey procedures and interview techniques prior to the study.

Based on the sample size estimation formula for stratified sampling (Formula 1), the minimum required sample size to achieve statistical significance was calculated using the 200 cases (*N*) obtained in the pilot survey, as follows:


$$n=\frac{Z_{\alpha /2}^{2}\pi (1-\pi )}{\delta^{2}}$$ (1)

$$\pi =\sum_{i=1}^{L}\pi_{i}N_{i}∕N$$ (2)

In the economically advantaged and disadvantaged areas (*L* = 2), 99 (*N*₁) and 101 (*N*₂) participants, respectively, were enrolled in the pilot survey. The weighted average of the stratum-specific proportions was calculated using Formula 2 to obtain *π*. With a two-sided significance level of *α* = 0.05 and an allowable error *δ* = 0.02, the required total sample size was estimated to be *n* = 865. Because this study involved a special population and relatively large sampling fluctuations were anticipated, we increased the target sample size to reduce the impact of uncontrollable factors during sampling and data collection and to ensure both an adequate questionnaire response rate and sufficient statistical power. In total, 1,111 postpartum women completed the SDS. After applying the predefined inclusion and exclusion criteria, 892 questionnaires were retained for the final analysis. Inclusion criteria were singleton pregnancy, provision of written informed consent, and willingness to comply with follow-up arrangements. Exclusion criteria were multiple pregnancy, pre-existing maternal health conditions (e.g., diagnosed psychiatric disorders), and refusal or inability to provide informed consent. Ethical approval for the study protocol was obtained from the Medical Research Ethics Committee of Chongqing Medical University before data collection began.

### 2.3 Study content and measurements

#### 2.3.1 Study content

In this study, the dependent variable is the occurrence of PPD. The independent variables include demographic characteristics (age, place of residence, education level, employment status, and monthly income in RMB), antenatal factors (exercise during pregnancy, number of antenatal education sessions, number of antenatal visits, antenatal examination for abnormalities, and inpatient environment), delivery-related factors (delivery method, knowledge level of delivery), and characteristics of mental health and social support (family care, social support, pregnancy-related stress, pregnancy depression, and pregnancy anxiety).

#### 2.3.2 Knowledge level of delivery

Participants’ knowledge of delivery modes was assessed using a self-developed 17-item questionnaire, in which each item was scored as 2 points for a correct answer, 1 point for an uncertain response, and 0 points for an incorrect answer. Total scores were classified into three levels of knowledge based on percentage thresholds, with scores below 60% indicating low knowledge, 60–80% indicating moderate knowledge, and above 80% indicating high knowledge; correspondingly, knowledge levels were categorized as 0–20 for low, 21–27 for moderate, and 28–34 for high. The internal consistency of this scale, as measured by Cronbach’s alpha, was 0.748 in the present study.

#### 2.3.3 Family and social factors

The Family Adaptation Partnership Growth Affection and Resolve (APGAR) scale (16) was used in this study to assess postpartum women’s overall satisfaction with family care. This scale consists of five items covering adaptation, partnership, growth, affection, and intimacy. Each item is scored from 0 to 2, and the total score is calculated as the sum of all item scored. A total score of 0–3 indicates severe family dysfunction, 4–6 indicates moderate dysfunction, and 7–10 indicates good family functioning (17). In this study, Cronbach’s alpha for the family functioning scale was 0.848.

The Social Support Rating Scale (SSRS), developed by Xiao Shuiyuan in 1986 (18), was used to assess the degree of psychological and practical support received by postpartum women in their social lives and their utilization of such support. The scale comprises 10 items across three dimensions: subjective support, objective support, and utilization of social support. Items 1–5 and 8–10 are rated on a 1–4 scale, whereas items 6 and 7 are scored based on the number of available sources of support, with 0 indicating no sources of support and multiple sources yielding higher scores. The total score is the sum of all item scores. A total score <35 indicates a low level of social support, a score of 35–45 indicates a moderate level, and a score >45 indicates a high level of social support (17). In this study, Cronbach’s alpha for the SSRS was 0.791.

#### 2.3.4 Depression measurement

Perinatal depression was measured using the Self-Rating Depression Scale (SDS) (19). The scale consists of 10 items, each rated on a 1–4 scale. The Depression Severity Index (DSI) was calculated by dividing the total score by 40. A DSI < 0.50 indicates no depression, 0.50–0.59 indicates mild depression, 0.60–0.69 indicates moderate depression, and ≥0.70 indicates severe depression. In this study, DSI ≥ 0.50 was considered indicative of the presence of depression. Cronbach’s alpha for the SDS in the postpartum period was 0.892.

### 2.4 Statistical analysis

Statistical analyses were performed using R software version 4.3.3. First, the baseline characteristics of the 892 participants were described and compared. The study population was then randomly divided into a training set and a validation set in a 7:3 ratio. Univariate logistic regression was conducted in the training set, and variables with a *p-value* of <0.05 were included in the multivariable logistic regression analysis. Stepwise regression with a selection criterion of *p* < 0.05 was used to identify factors associated with PPD and to develop the risk prediction model. The model’s discrimination was evaluated using the area under the receiver operating characteristic (ROC) curve (AUC), with a larger AUC indicating better discriminative ability. ROC curves were plotted for both the training and validation sets. Calibration curves were used to assess the agreement between predicted and observed probabilities, and predictive performance in both sets was evaluated in terms of accuracy, discrimination, and calibration to ensure the model’s reliability and generalizability. Finally, decision curve analysis (DCA) was performed to assess the clinical utility of the model.

## 3 Results

### 3.1 Descriptive statistics and reliability of scales

Overall, participants demonstrated a moderate level of delivery-related knowledge, with a mean score of 21.26 ± 5.81 on the self-developed questionnaire; however, 42.38% of participants had a low level of delivery-related knowledge. The mean normalized score of the SDS was 0.33 ± 0.10, indicating an overall absence of depressive symptoms in the sample. Family care, as measured by the Family APGAR scale, showed a mean score of 7.71 ± 2.37, reflecting generally good family care, although 28.03% of participants reported moderate to severe family dysfunction. Social support levels were moderate, with an average SSRS score of 38.85 ± 7.71, while 28.36% of participants reported low levels of social support. All four scales demonstrated acceptable to high internal consistency, with Cronbach’s *α* values ranging from 0.748 to 0.892. The detailed results are provided in Table 1.

**Table 1:** Descriptive statistics and reliability of scales.

Scale	Score range	Cutoff categories	*N* (%)	Score (mean ± SD)	Cronbach’ s *α*
Knowledge level of delivery (self-designed)	0–34	0–20 (Low) 21–27 (Moderate) 28–34 (High)	378 (42.38%) 391 (43.83%) 123 (13.79%)	21.26 ± 5.81	0.748
Self-Rating Depression Scale (SDS)	0.25–1	<0.50 (No depression) ≥0.50 (Depression)	801 (89.8%) 91 (10.2%)	0.33 ± 0.10	0.892
Family APGAR	0–10	0–3 (Severe) 4–6 (Moderate) 7–10 (Good functioning)	50 (5.61%) 200 (22.42%) 642 (71.97%)	7.71 ± 2.37	0.848
Social Support Rating Scale (SSRS)	0–50	<35 (Low) 35–45 (Moderate) >45 (High)	253 (28.36%) 464 (52.02%) 175 (19.62%)	38.85 ± 7.71	0.791

### 3.2 Participants’ characteristics

This study included 892 postpartum women, of whom 91 (10.2%) were diagnosed with PPD. Univariate analysis showed no significant differences between the PPD and non-PPD groups in terms of age, place of residence, employment status, monthly income, number of antenatal education sessions, or antenatal examinations (*p* > 0.05). However, significant differences were observed in educational level, exercise during pregnancy, number of antenatal tests, inpatient environment, delivery-related knowledge, family care, social support, and mode of delivery (*p* < 0.05). Specifically, the prevalence of PPD was higher among women with lower educational levels, those who exercised during pregnancy, those with fewer antenatal visits, those reporting a less favorable inpatient environment, those with lower delivery-related knowledge, those reporting family dysfunction, those with low social support, and those who delivered by cesarean section, as shown in Table 2.

**Table 2:** Baseline characteristics of participants.

Characteristics	Total (*N* = 892)	Non-depression (*N* = 801)	Depression (*N* = 91)	*P*
Demographic characteristics				
Age, years, *n* (%)				0.767
>30	38 (4.26)	34 (4.24)	4 (4.40)	
25–30	473 (53.03)	428 (53.43)	45 (49.45)	
16–24	381 (42.71)	339 (42.32)	42 (46.15)	
Place of residence, *n* (%)				0.380
Rural	354 (39.69)	314 (39.20)	40 (43.96)	
Urban	538 (60.31)	487 (60.80)	51 (56.04)	
Education level, *n* (%)				0.046*
College education or above	367 (41.14)	338 (42.20)	29 (31.87)	
Senior high school	267 (29.93)	241 (30.09)	26 (28.57)	
Junior high school or below	258 (28.92)	222 (27.72)	36 (39.56)	
Working status, *n* (%)				0.562
Employed	457 (51.23)	413 (51.56)	44 (48.35)	
Housewife/unemployed	435 (48.77)	388 (48.44)	47 (51.65)	
Monthly income (RMB), *n* (%)				0.463
≥5,001	304 (34.08)	275 (34.33)	29 (31.87)	
3,001–5,000	379 (42.49)	335 (41.82)	44 (48.35)	
≤3,000	209 (23.43)	191 (23.85)	18 (19.78)	
Antenatal factors				
Exercise during pregnancy, *n* (%)				0.017*
Yes	674 (75.56)	596 (74.41)	78 (85.71)	
No	218 (24.44)	205 (25.59)	13 (14.29)	
Number of antenatal education in pregnancy, *n* (%)				0.483
0	336 (37.67)	307 (38.33)	29 (31.87)	
1–5	330 (37.00)	293 (36.58)	37 (40.66)	
≥6	226 (25.34)	201 (25.09)	25 (27.47)	
Number of antenatal tests, *n* (%)				0.014*
≥11	437 (48.99)	404 (50.44)	33 (36.26)	
6–10	386 (43.27)	340 (42.45)	46 (50.55)	
<6	69 (7.74)	57 (7.12)	12 (13.19)	
Antenatal examination for abnormalities, *n* (%)				0.697
No	752 (84.30)	674 (84.14)	78 (85.71)	
Yes	140 (15.70)	127 (15.86)	13 (14.29)	
Inpatient environment, *n* (%)				<0.001**
Very good	417 (46.75)	390 (48.69)	27 (29.67)	
Quite good	350 (39.24)	307 (38.33)	43 (47.25)	
Average	125 (14.01)	104 (12.98)	21 (23.08)	
Delivery situation				
Delivery method, *n* (%)				0.005**
Vaginal delivery	588 (65.92)	540 (67.42)	48 (52.75)	
Cesarean section	304 (34.08)	261 (32.58)	43 (47.25)	
Knowledge level of delivery, *n* (%)				<0.001**
High	236 (26.46)	228 (28.46)	8 (8.79)	
Moderate	446 (50.00)	414 (51.69)	32 (35.16)	
Low	210 (23.54)	159 (19.85)	51 (56.04)	
Social support characteristics				
Family care, *n* (%)				<0.001**
Family functioning is within normal limits	645 (72.31)	619 (77.28)	26 (28.57)	
Moderate dysfunction in family functioning	198 (22.20)	146 (18.23)	52 (57.14)	
Severe dysfunction in family functioning	49 (5.49)	36 (4.49)	13 (14.29)	
Social support, *n* (%)				<0.001**
High	175 (19.62)	168 (20.97)	7 (7.69)	
Moderate	472 (52.91)	426 (53.18)	46 (50.55)	
Low	245 (27.47)	207 (25.84)	38 (41.76)	

The symbol “*” indicates *p* < 0.05, and “**” represents *p* < 0.01.

### 3.3 Analysis of differences among variables between training and validation sets

All 892 postpartum women included in this study were randomly allocated to the training and validation sets in a 7:3 ratio, with stratification by depression status. The training set comprised 624 women (67 with PPD and 557 without PPD), whereas the validation set comprised 268 women (24 with PPD and 244 without PPD). Baseline characteristics were compared between the two sets using chi-square tests and non-parametric methods (including rank-sum tests for ordinal variables). No statistically significant differences were observed between the training and validation sets in terms of sociodemographic characteristics, antenatal factors, delivery conditions, psychological health, or social support (all *p* > 0.05). These findings indicate that the two datasets were well balanced and homogeneous across all measured variables, as shown in Supplementary Table S1.

### 3.4 Univariate logistic regression analysis of the training set samples

Univariate logistic regression analysis was conducted in the training set of 624 postpartum women (Table 3). No statistically significant associations with PPD were observed for maternal age, place of residence, educational level, employment status, monthly income, exercise during pregnancy, number of antenatal education sessions, or abnormal findings during antenatal examinations (all *p* > 0.05). In contrast, significant associations were found for the number of antenatal visits, inpatient environment, mode of delivery, delivery-related knowledge level, family care, and social support (all *p* < 0.05). Compared with their respective reference groups, women who had fewer than six antenatal visits (odds ratio (OR) = 2.54, 95% confidence interval (95% CI): 1.11–5.80), reported an average-quality inpatient environment (3.09, 1.60–5.95), or delivered by cesarean section (2.15, 1.29–3.59) had higher odds of PPD. Similarly, women with moderate (2.47, 1.00–6.11) or low (9.18, 3.72–22.65) delivery-related knowledge, moderate (8.39, 4.65–15.13) or severe (9.64, 4.16–22.29) family dysfunction, and moderate (4.95, 1.50–16.33) or low (6.85, 2.02–23.25) social support had significantly increased odds of developing PPD.

**Table 3:** Univariate logistic regression analysis of training set samples.

Characteristics	β	SE	Z	*P*	OR (95% CI)
Demographic characteristics					
Age, years					
>30					1.00 (Reference)
25–30	0.05	0.63	0.08	0.933	1.05 (0.30 ~ 3.66)
16–24	0.20	0.64	0.31	0.754	1.22 (0.35 ~ 4.25)
Place of residence					
Rural					1.00 (Reference)
Urban	0.04	0.27	0.14	0.890	1.04 (0.62 ~ 1.74)
Education level					
College education or above					1.00 (Reference)
Senior high school	−0.13	0.33	−0.41	0.685	0.88 (0.46 ~ 1.66)
Junior high school or below	0.30	0.30	0.99	0.324	1.35 (0.75 ~ 2.43)
Working status					
Employed					1.00 (Reference)
Housewife/unemployed	0.11	0.26	0.41	0.684	1.11 (0.67 ~ 1.84)
Monthly income (RMB)					
≥5,001					1.00 (Reference)
3,001–5,000	0.14	0.29	0.48	0.633	1.15 (0.65 ~ 2.04)
≤3,000	−0.15	0.36	−0.40	0.687	0.86 (0.42 ~ 1.76)
Antenatal factors					
Exercise during pregnancy					
Yes					1.00 (Reference)
No	−0.50	0.33	−1.49	0.136	0.61 (0.32 ~ 1.17)
Number of antenatal education sessions in pregnancy					
0					1.00 (Reference)
1–5	0.31	0.31	1.00	0.320	1.36 (0.74 ~ 2.49)
≥6	0.48	0.33	1.46	0.145	1.62 (0.85 ~ 3.10)
Number of antenatal tests					
≥11					1.00 (Reference)
6–10	0.25	0.28	0.90	0.367	1.28 (0.75 ~ 2.21)
<6	0.93	0.42	2.20	0.027*	2.54 (1.11 ~ 5.80)
Antenatal examination for abnormalities					
No					1.00 (Reference)
Yes	0.12	0.34	0.37	0.714	1.13 (0.58 ~ 2.20)
Inpatient environment					
Very good					1.00 (Reference)
Quite good	0.28	0.30	0.93	0.352	1.33 (0.73 ~ 2.40)
Average	1.13	0.33	3.37	<0.001**	3.09 (1.60 ~ 5.95)
Delivery situation					
Delivery method					
Vaginal delivery					1.00 (Reference)
Cesarean section	0.77	0.26	2.94	0.003**	2.15 (1.29 ~ 3.59)
Knowledge level of delivery					
High					1.00 (Reference)
Moderate	0.90	0.46	1.96	0.050	2.47 (1.00 ~ 6.11)
Low	2.22	0.46	4.81	<0.001**	9.18 (3.72 ~ 22.65)
Social support characteristics					
Family care					
Family functioning is within normal limits					1.00 (Reference)
Moderate dysfunction in family functioning	2.13	0.30	7.06	<0.001**	8.39 (4.65 ~ 15.13)
Severe dysfunction in family functioning	2.27	0.43	5.29	<0.001**	9.64 (4.16 ~ 22.29)
Social support					
High					1.00 (Reference)
Moderate	1.60	0.61	2.63	0.009**	4.95 (1.50 ~ 16.33)
Low	1.92	0.62	3.08	0.002**	6.85 (2.02 ~ 23.25)

OR, odds ratio. 95% CI, 95% confidence interval. The symbol “*” indicates *p* < 0.05, and “**” represents *p* < 0.01.

### 3.5 Development and performance evaluation of the predictive model

**Figure 1:**
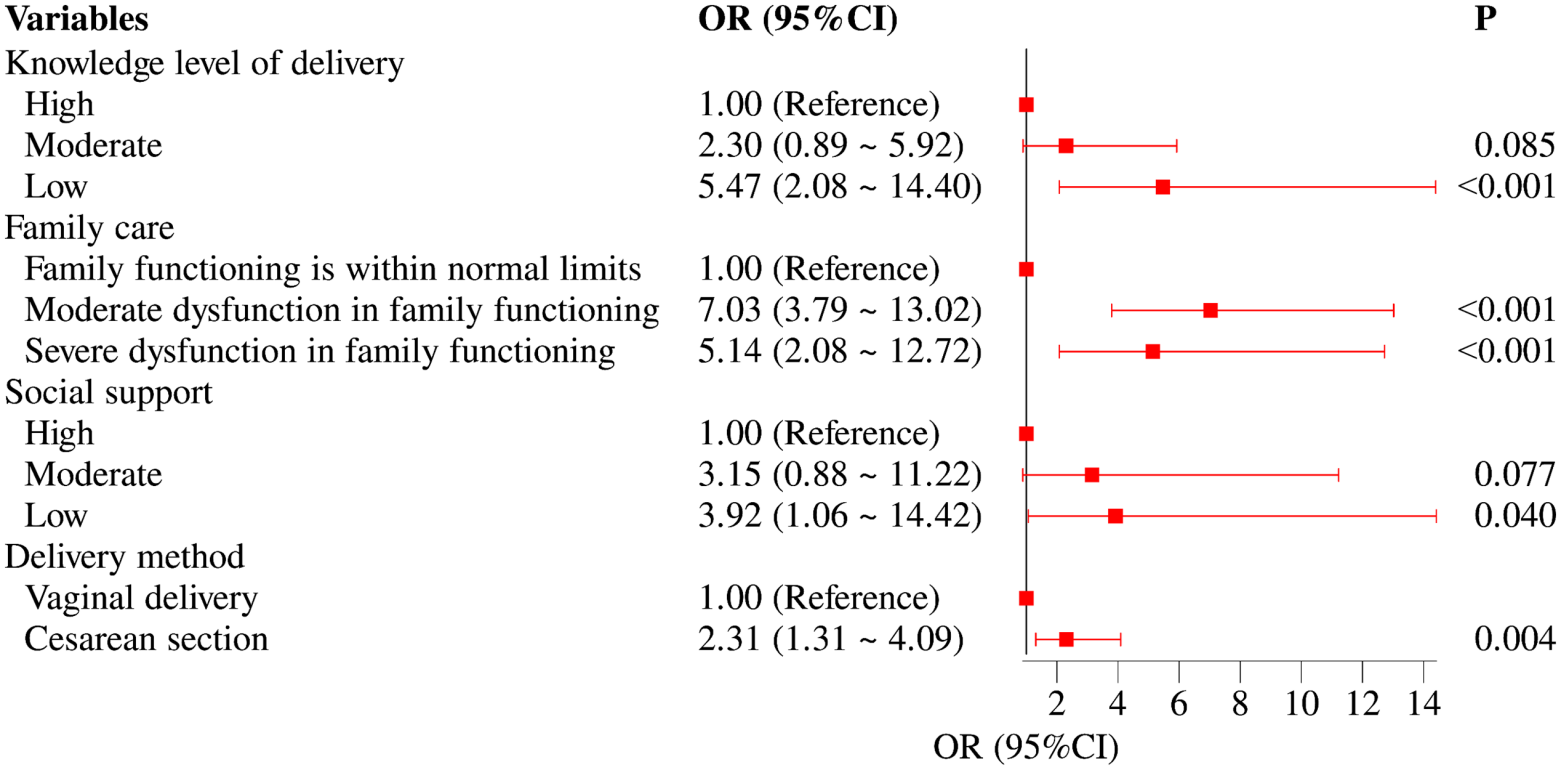
Forest plot of multi-factor logistic regression analysis.

A multivariable logistic regression analysis was performed to further examine the variables identified as significant in the univariate analysis, using stepwise selection with entry and removal criteria of *p* = 0.05. As illustrated in the forest plot (Figure 1), four factors were independently associated with PPD: delivery-related knowledge, family care, social support, and mode of delivery. Women with low delivery-related knowledge had a markedly higher risk of developing PPD than those with moderate or high knowledge (*p* < 0.001; OR = 5.47, 95% CI: 2.08–14.40). Women with moderate (7.03, 3.79–13.02) or severe (5.14, 2.08–12.72) family dysfunction were also significantly more likely to develop PPD than those with normal family functioning (both *p* < 0.001). Low social support was a strong predictor, increasing the odds of PPD by approximately 4-fold compared with high social support (0.040; 3.92, 1.06–14.42). In addition, women who delivered by cesarean section had more than twice the odds of PPD compared with those who had vaginal deliveries (0.004; 2.31, 1.31–4.09). The variables retained in the final predictive model were low delivery-related knowledge, moderate and severe family dysfunction, low social support, and cesarean section. The final predictive equation for PPD was logit(P) = −5.38 + 1.70 × (low delivery-related knowledge) + 1.95 × (moderate family dysfunction) + 198 1.64 × (severe family dysfunction) + 1.36 × (low social support) + 0.84 × (cesarean section), as shown in Supplementary Table S2.

**Figure 2:**
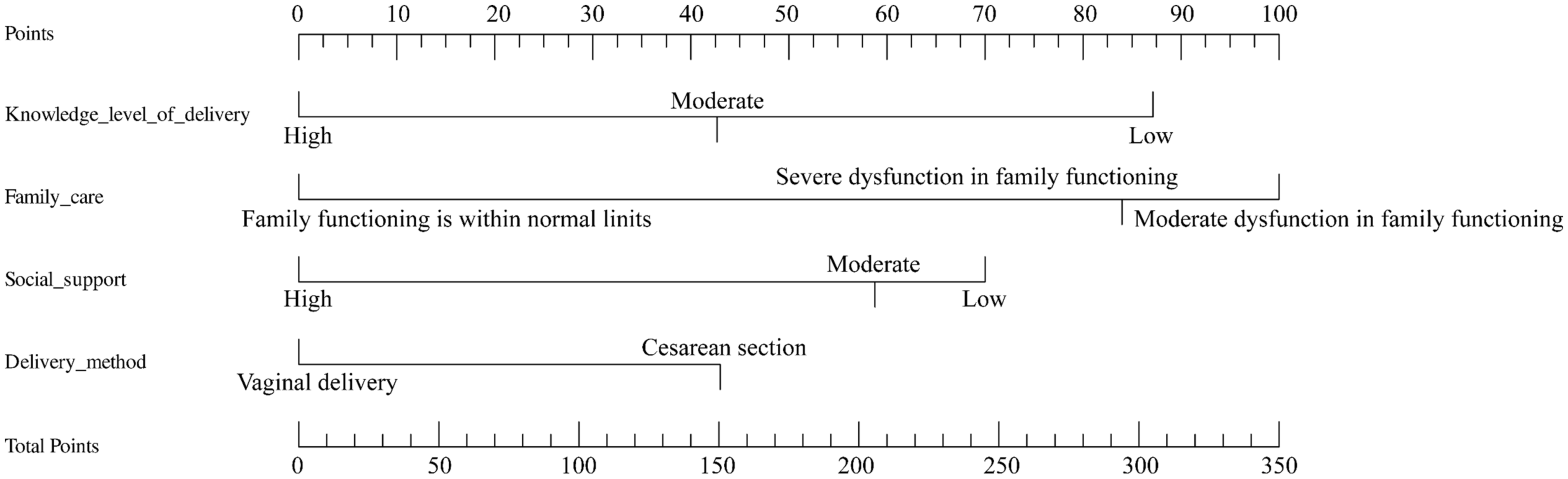
Nomogram integrates four predictors: knowledge level of delivery, family care, social support, and delivery method. To use the nomogram, locate the patient’s value for each predictor and draw a vertical line upward to the “Points” scale to determine the score. The scores for all predictors are summed to obtain the “Total Points,” which is then projected downward to the “Risk” scale to estimate the individual probability of postpartum depression (PPD).

Based on the independent predictors identified in the multivariable logistic regression analysis, we constructed a nomogram to predict the risk of PPD (Figure 2). The nomogram converts the regression coefficients of each predictor into an intuitive point-based scoring system. Clinicians can locate an individual woman’s values (e.g., delivery-related knowledge level and family functioning status) on the corresponding axes, assign the associated number of points, sum these to obtain a total score, and then project this total onto the bottom scale to estimate her probability of PPD. This tool enables visual, individualized risk assessment and provides an intuitive aid to subsequent clinical decision-making.

**Figure 3:**
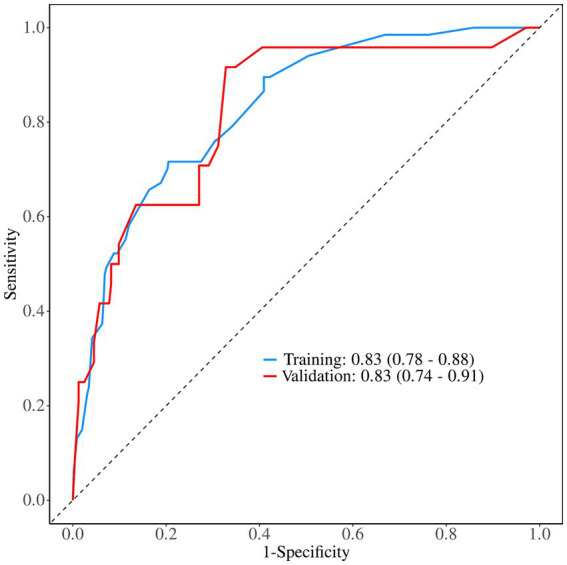
Comparison of the area under the ROC curve between the training and validation sets.

**Figure 4:**
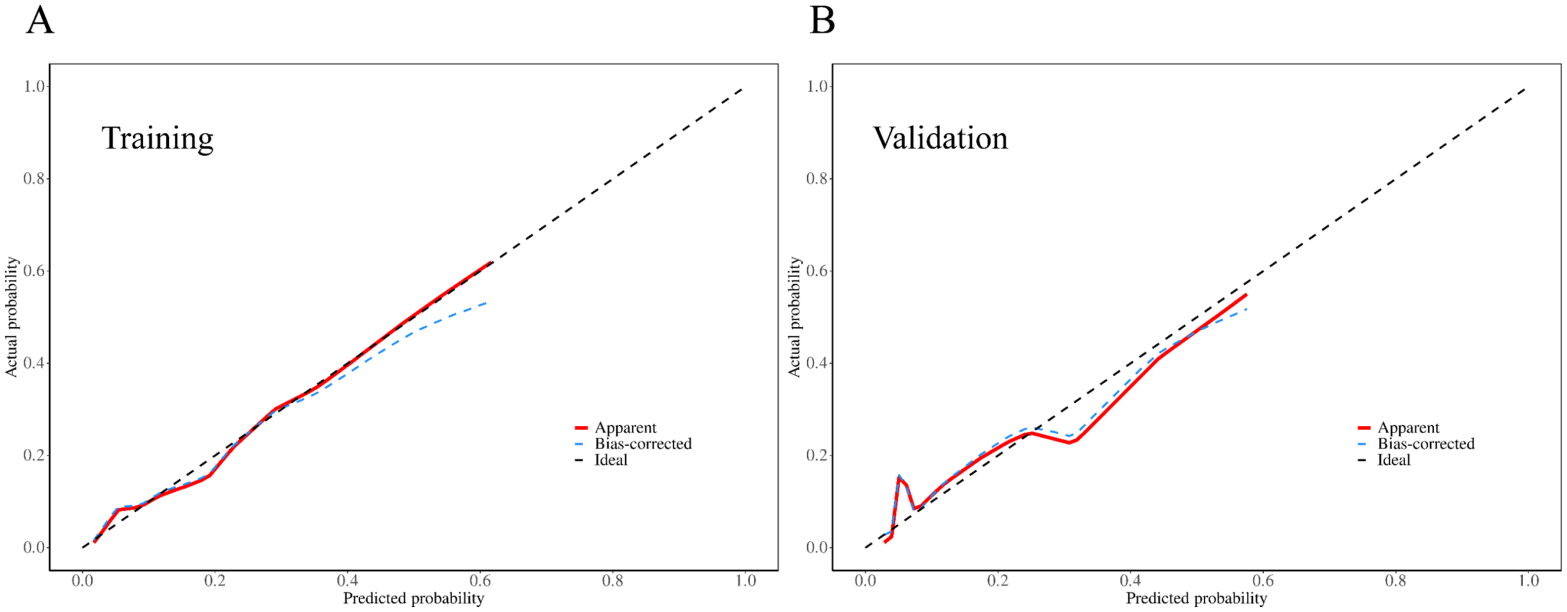
Calibration curves of the predictive nomogram. **(A)** Calibration curve for the training cohort. **(B)** Calibration curve for the validation cohort.

The discriminative performance of the model was evaluated using the AUC. In the training set, the AUC was 0.83 (95% CI: 0.78–0.88), and the validation set demonstrated a comparable AUC of 0.83 (95% CI: 0.74–0.91), indicating good and generalizable discriminative ability (Figure 3). Calibration curves for both the training and validation sets showed good agreement between predicted probabilities and observed outcomes, indicating satisfactory model calibration (Figure 4).

Specific performance metrics are presented in Supplementary Table S3. In the training set, the model had an accuracy of 0.80, a sensitivity of 0.72, a specificity of 0.96, a positive predictive value (PPV) of 0.30, and a negative predictive value (NPV) of 0.96. In the validation set, the corresponding metrics were an accuracy of 0.73, a sensitivity of 0.71, a specificity of 0.96, a PPV of 0.20, and an NPV of 0.90.

**Figure 5:**
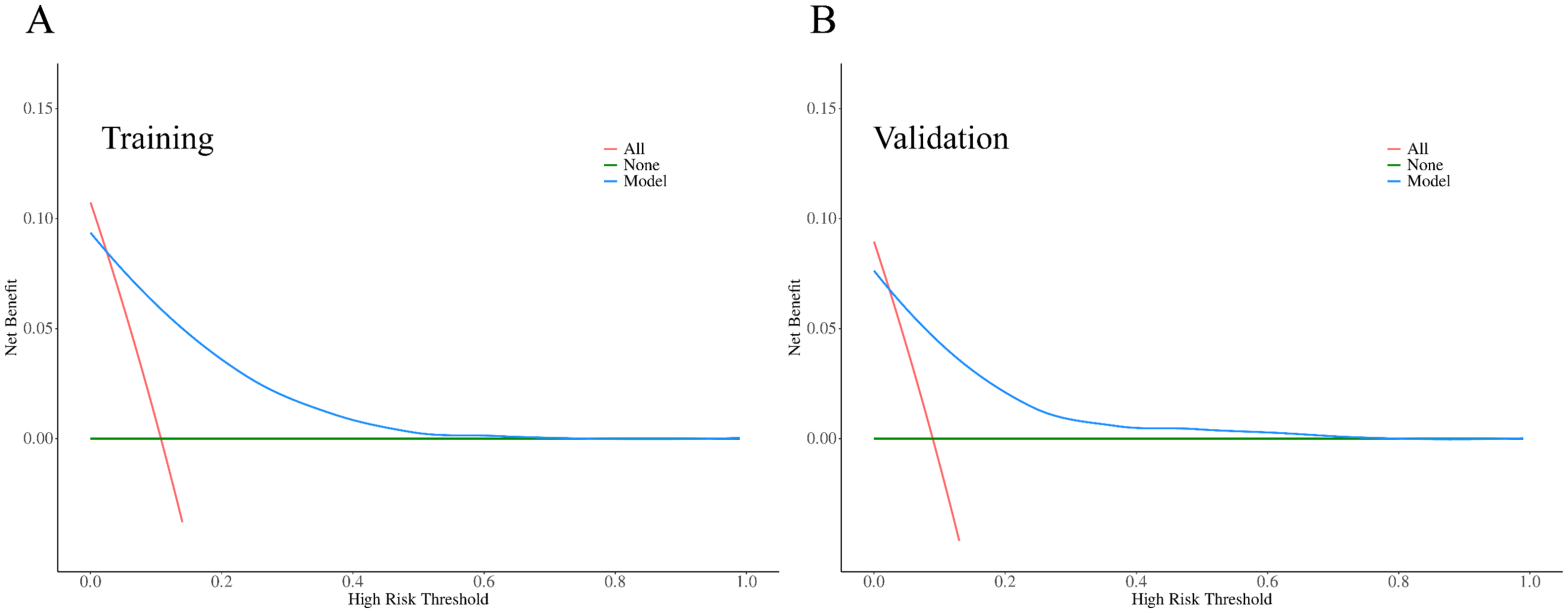
Decision curve analysis (DCA) for the predictive model **(A)** DCA of the training set and **(B)** DCA of the validation set.

Decision curve analysis (DCA) was used to evaluate the clinical utility of the model. As shown in Figure 5, the net benefit curves for both the training and validation sets lie above the “treat-all” and “treat-none” reference lines across a wide threshold probability range of 10% to 70%. This indicates that using the predictive model for clinical decision-making provides greater net benefit than either universal intervention or no intervention within this range. Beyond this interval, the net benefit of the model decreased and gradually converged with that of the “treat-none” strategy. Taken together, these findings suggest that the model has favorable and stable clinical utility for threshold probabilities between 10 and 70%.

## 4 Discussion

### 4.1 Prevalence of postpartum depression and model performance

In this cross-sectional investigation conducted among 892 postpartum women in Chongqing, China, we identified several psychosocial and obstetric factors that independently predicted postpartum depression (PPD) and developed a robust predictive model. The prevalence of PPD in this cohort was 10.2%, which is consistent with recent multinational studies reporting a pooled prevalence of 13.6% (12). This convergence across studies may reflect shared postpartum challenges despite differences in sociocultural settings. However, some methodological variations should be noted. The multinational study primarily relied on the Edinburgh Postnatal Depression Scale (EPDS), whereas our study used the Self-Rating Depression Scale (SDS), and differences in screening instruments may influence the identification of depressive symptoms. Additionally, cultural norms in China, such as structured postpartum care and strong family involvement, could shape symptom expression and help-seeking patterns, potentially affecting the measured prevalence. Taken together, the similarity in prevalence across culturally diverse samples strengthens the external validity of our findings, while any discrepancies may be attributed to differences in measurement approaches and contextual factors. Multivariable logistic regression revealed that low delivery-related knowledge (OR = 5.47, 95% CI: 2.08–14.40), moderate family dysfunction (7.03, 3.79–13.02), severe family dysfunction (5.14, 2.08–12.72), low social support (3.92, 1.06–14.42), and cesarean section (2.31, 1.31–4.09) were independent predictors of PPD. Based on these variables, we developed a logistic regression model and constructed a nomogram to facilitate clinical application. The model demonstrated strong discriminatory power (AUC = 0.83 in both training and validation datasets) and good calibration, with decision curve analysis supporting its potential utility as an early-warning tool in public health and clinical practice to reduce the adverse consequences of PPD for mothers and their families.

### 4.2 Family functioning and social support

Family dysfunction emerged as the strongest predictor. Interpersonal conflicts, low emotional support, or insufficient practical assistance within the family environment may undermine maternal coping capacity and increase psychological vulnerability. Well-functioning families provide stable emotional bonds, effective resource sharing, and constructive communication, which buffer maternal stress associated with parenting demands, physical discomfort, and role transitions. In contrast, dysfunctional family interactions may trigger feelings of loneliness, helplessness, and frustration, consistent with previous research (20), activate the hypothalamic–pituitary–adrenal (HPA) axis, elevate cortisol levels, disrupt emotional regulation, and increase the risk of depression (21). Low social support is also a significant predictor of PPD. Inadequate support from partners, relatives, or the community can exacerbate maternal isolation, reduce coping capacity, and limit access to emotional and instrumental resources, leading to depressive symptoms through the activation of the HPA axis and elevated cortisol levels. Conversely, adequate social support serves as both an emotional buffer and a source of practical assistance, enhancing self-efficacy and psychological resilience, mitigating stress and anxiety, and thereby reducing the likelihood of depressive symptoms, consistent with previous research (22). Therefore, enhancing family-based and community mental health interventions is critical. Developing programs that encourage family members to actively participate in the emotional and physical care of new mothers can provide a vital support system. Strengthening community mental health resources, such as peer support groups and counseling services, can also provide emotional support to mothers, especially in areas with fewer healthcare resources. From a public health perspective, integrating family-based mental health education and community support programs into existing maternal and child healthcare systems may improve early detection and management of PPD.

### 4.3 Delivery-related knowledge and cesarean section

Insufficient delivery-related knowledge is associated with an elevated risk of PPD (23, 24). Women who possess a comprehensive understanding of labor and potential complications typically exhibit greater perceived control, superior psychological preparedness, stronger self-efficacy, and more effective coping, facilitating adaptive emotion regulation during childbirth and postpartum role transitions. By contrast, knowledge deficits may heighten uncertainty and anxiety, amplify stress responses, and erode psychological resilience, thereby exacerbating depressive symptomatology (25, 26). Although direct empirical evidence remains limited, plausible mechanisms include activation of stress-response systems with concomitant elevations in cortisol, as well as reductions in self-efficacy that leave women feeling powerless when confronting the challenges of labor (21, 27, 28).

Cesarean section, as an obstetric factor, also significantly contributes to PPD. Recent meta-analyses indicate a modest but consistent increase in PPD risk following CS (29, 30). Women undergoing cesarean sections often face prolonged recovery, a higher risk of complications, increased pain and physical discomfort (31, 32), and limited early mother–infant contact (33, 34). Additionally, cesarean section may be associated with psychological distress, such as deviations from expected birth experiences or perceived loss of control (35, 36). These factors collectively can trigger stress responses, impair emotional regulation, and reduce psychological resilience, thereby elevating the risk of PPD. Therefore, targeted psychological support is also essential, particularly for women who have undergone cesarean sections. Postpartum care programs should prioritize women after a cesarean section by incorporating targeted mental health screening and referral mechanisms into routine postnatal services.

### 4.4 Additional factors and sociocultural context

In addition to the independent predictors identified, several factors were noteworthy for their significance in univariate analyses but not in multivariate models, including education level, inpatient environment, and the number of antenatal visits. Lower educational attainment was associated with higher PPD risk in univariate analysis, yet this association lost significance after adjustment, suggesting that its effect may be mediated by factors such as delivery-related knowledge and social support (37).

Similarly, the inpatient environment showed a significant univariate association with PPD, but not after multivariate adjustment, indicating that psychosocial factors may mediate the relationship between hospital conditions and maternal mental health. Fewer antenatal visits were also linked to increased PPD risk in univariate analysis; however, this effect disappeared after adjustment, likely reflecting broader social or healthcare disadvantages, such as economic hardship or barriers to access, rather than an independent causal influence on PPD.

Several cultural and social factors in China also influence the experience of PPD. Confinement traditions such as “zuo yuezi,” which require new mothers to stay indoors and avoid certain activities for a month after delivery, play a significant role in shaping women’s postpartum experiences. While intended to promote physical recovery, these practices can inadvertently contribute to social isolation and emotional stress, potentially exacerbating the symptoms of PPD. Women adhering to these traditions may experience heightened feelings of loneliness and lack of autonomy, especially if they feel isolated from their social networks. Moreover, traditional family roles in China place significant expectations on women to quickly resume their roles as caregivers while also recovering physically and emotionally from childbirth. This pressure can create internalized guilt or shame if a woman struggles with her mental health, leading to a reluctance to seek help. In a society where women are often expected to demonstrate resilience, acknowledging the need for mental health support can feel stigmatizing. The stigma around mental illness in China, particularly postpartum depression, may discourage women from discussing their symptoms, leading them to suffer in silence. This is compounded by the strong value placed on family honor, where mental health challenges may be perceived as a personal or familial weakness. Social support plays a key role in maternal mental health. In China, social support can often come from family members, with mothers typically expected to receive help from their mothers-in-law, husbands, or other relatives. However, as China urbanizes and nuclear families become more common, many women may find themselves with limited access to familial support. The lack of a robust social safety net or professional mental health services in some areas further complicates this issue, making it crucial for healthcare systems to promote better integration of mental health resources into maternal care. Additionally, socioeconomic pressures, such as the financial burden of raising children and the demands of balancing work and family responsibilities, may increase the likelihood of PPD by amplifying stress and reducing a mother’s sense of control over her life.

### 4.5 Policy implications, strengths, and limitations

From a policy perspective, these findings highlight the need for integrated maternal mental health programs that incorporate psychosocial, obstetric, and cultural factors. Healthcare systems should ensure that routine screening for PPD is implemented across all postpartum clinics. Training healthcare providers to identify and address cultural and psychosocial factors—such as family dysfunction and traditional postpartum practices—can improve the accuracy of PPD diagnoses and lead to more effective interventions. Additionally, policies that support improved social support networks, such as community-based mental health services and family support programs, are crucial to reducing the burden of PPD.

This study has several strengths, including a relatively large sample size, the integration of psychosocial and clinical factors, and the clear distinction between univariate and multivariate predictors. However, there are several limitations that should be acknowledged. First, the cross-sectional design of the study precludes causal inference, as it only captures associations at a single point in time. Consequently, we cannot conclude the directionality or temporality of the observed relationships. Second, the study relied on self-reported data, which may introduce recall bias and social desirability bias, affecting the accuracy of the responses. Participants may have over- or under-reported certain behaviors or experiences due to personal perceptions or social influences. Third, the data used in this study were collected at a single time point and may not fully reflect current postpartum depression trends, as the dataset may be somewhat outdated. Therefore, the results may not entirely represent the most up-to-date prevalence or risk factors. Finally, the generalizability of our findings is limited, as the sample may not be fully representative of broader populations. Cultural and contextual factors, such as differences in healthcare access or postpartum care practices, may affect the applicability of our findings to other settings or populations. Additionally, variables such as pre-existing mental health conditions or detailed socioeconomic factors were not fully accounted for, which may lead to residual confounding.

## 5 Conclusion

This study identified low delivery-related knowledge, family dysfunction, low social support, and cesarean section as independent predictors of PPD in women in Chongqing, China. The resulting predictive model exhibited strong discrimination and calibration, supporting its potential as an early-warning tool for clinical use. By enabling the timely identification of high-risk individuals, the model provides an empirical basis for targeted interventions to improve maternal mental health. Further longitudinal and multicenter studies are needed to validate these findings, explore the underlying mechanisms, and develop scalable prevention strategies.

## Data Availability

The raw data supporting the conclusions of this article will be made available by the authors, without undue reservation.
