# Supplementary material for: Risk factors and predictive modeling of postpartum depression among postpartum women: empirical evidence from Chongqing, China
Source: Front Public Health. 2026 Feb 13;14:1725970. doi: 10.3389/fpubh.2026.1725970 (PMC12945766; doi:10.3389/fpubh.2026.1725970)
Supplement: Supplementary file 1 [file Data_Sheet_1.pdf]

Table S1. Analysis of variable differences between the training set and the validation set.

Characteristics	Total (N=892)	Validation set (N=268)	Training set (N=624)	Statistic	*P*
**Demographic characteristics**					
Age, years, n (%)				χ^2=2.82	0.244
>30	38 (4.26)	7 (2.61)	31 (4.97)		
25-30	473 (53.03)	148 (55.22)	325 (52.08)		
16-24	381 (42.71)	113 (42.16)	268 (42.95)		
Place of residence, n (%)				χ^2=0.01	0.924
Rural	354 (39.69)	107 (39.93)	247 (39.58)		
Urban	538 (60.31)	161 (60.07)	377 (60.42)		
Education level, n (%)				χ^2=0.07	0.965
College education or above	367 (41.14)	112 (41.79)	255 (40.87)		
Senior high school	267 (29.93)	79 (29.48)	188 (30.13)		
Junior high school or below	258 (28.92)	77 (28.73)	181 (29.01)		
Working status, n (%)				χ^2=0.11	0.736
Employed	457 (51.23)	135 (50.37)	322 (51.60)		
Housewife/Unemployed	435 (48.77)	133 (49.63)	302 (48.40)		
Monthly income (RMB), n (%)				χ^2=1.37	0.505
≥5001	304 (34.08)	84 (31.34)	220 (35.26)		
3001-5000	379 (42.49)	117 (43.66)	262 (41.99)		

Characteristics	Total (N=892)	Validation set (N=268)	Training set (N=624)	Statistic	*P*
≤3000	209 (23.43)	67 (25.00)	142 (22.76)		
**Antenatal factors**					
Exercise during pregnancy, n (%)				χ^2=1.22	0.269
Yes	674 (75.56)	209 (77.99)	465 (74.52)		
No	218 (24.44)	59 (22.01)	159 (25.48)		
Number of antenatal education in pregnancy, n (%)				χ^2=2.24	0.326
0	336 (37.67)	92 (34.33)	244 (39.10)		
1-5	330 (37.00)	101 (37.69)	229 (36.70)		
≥6	226 (25.34)	75 (27.99)	151 (24.20)		
Number of antenatal tests, n (%)				χ^2=1.28	0.527
≥11	437 (48.99)	125 (46.64)	312 (50.00)		
6-10	386 (43.27)	119 (44.40)	267 (42.79)		
<6	69 (7.74)	24 (8.96)	45 (7.21)		
Antenatal examination for abnormalities, n (%)				χ^2=0.67	0.415
No	752 (84.30)	230 (85.82)	522 (83.65)		
Yes	140 (15.70)	38 (14.18)	102 (16.35)		
Inpatient environment, n (%)				χ^2=3.14	0.208
Very good	417 (46.75)	116 (43.28)	301 (48.24)		
Quite good	350 (39.24)	117 (43.66)	233 (37.34)		

Characteristics	Total (N=892)	Validation set (N=268)	Training set (N=624)	Statistic	*P*
Average	125 (14.01)	35 (13.06)	90 (14.42)		
**Delivery situation**					
Delivery method, n (%)				χ^2=1.05	0.305
Vaginal delivery	588 (65.92)	170 (63.43)	418 (66.99)		
Cesarean section	304 (34.08)	98 (36.57)	206 (33.01)		
Knowledge level of delivery, n (%)				χ^2=5.01	0.082
High	236 (26.46)	68 (25.37)	168 (26.92)		
Moderate	446 (50.00)	124 (46.27)	322 (51.60)		
Low	210 (23.54)	76 (28.36)	134 (21.47)		
**Social support characteristics**					
Family care, n (%)				χ^2=2.37	0.306
Family functioning is within normal limits	645 (72.31)	187 (69.78)	458 (73.40)		
Moderate dysfunction in family functioning	198 (22.20)	68 (25.37)	130 (20.83)		
Severe dysfunction in family functioning	49 (5.49)	13 (4.85)	36 (5.77)		
Social support, n (%)				χ^2=2.50	0.286
High	175 (19.62)	57 (21.27)	118 (18.91)		
Moderate	472 (52.91)	131 (48.88)	341 (54.65)		
Low	245 (27.47)	80 (29.85)	165 (26.44)		
Depression, n (%)				χ^2=0.65	0.420

Characteristics	Total (N=892)	Validation set (N=268)	Training set (N=624)	Statistic	*P*
No	801 (89.80)	244 (91.04)	557 (89.26)		
Yes	91 (10.20)	24 (8.96)	67 (10.74)		

Table S2. Coefficients of variables in the predictive model.

Characteristics	β	S.E	Z	*P*	OR (95%CI)
Intercept	-5.38	0.77	-7.00	<.001**	0.00 (0.00 ~ 0.02)
Knowledge level of delivery					
High					1.00 (Reference)
Moderate	0.83	0.48	1.72	0.085	2.30 (0.89 ~ 5.92)
Low	1.70	0.49	3.44	<.001**	5.47 (2.08 ~ 14.40)
Family care					
Family functioning is within normal limits					1.00 (Reference)
Moderate dysfunction in family functioning	1.95	0.31	6.20	<.001**	7.03 (3.79 ~ 13.02)
Severe dysfunction in family functioning	1.64	0.46	3.54	<.001**	5.14 (2.08 ~ 12.72)
Social support					
High					1.00 (Reference)
Moderate	1.15	0.65	1.77	0.077	3.15 (0.88 ~ 11.22)
Low	1.36	0.67	2.05	0.040*	3.92 (1.06 ~ 14.42)

Characteristics	β	S.E	Z	*P*	OR (95%CI)
Delivery method					
Vaginal delivery					1.00 (Reference)
Cesarean section	0.84	0.29	2.88	0.004**	2.31 (1.31 ~ 4.09)

β: standardized regression coefficients. OR: Odds Ratio, CI: Confidence Interval. The symbol "*" indicates $p < 0.05$, and "**" represents $p < 0.01$.

Table S3. Confusion matrix (combined training + validation set).

	AUC (95%CI)	Accuracy (95%CI)	Sensitivity (95%CI)	Specificity (95%CI)	PPV (95%CI)	NPV (95%CI)
Train	0.83 (0.78-0.88)	0.79 (0.75-0.82)	0.80 (0.76-0.83)	0.72 (0.61-0.82)	0.96 (0.94-0.98)	0.30 (0.23-0.37)
Test	0.83 (0.74-0.91)	0.72 (0.67-0.78)	0.73 (0.67-0.78)	0.71 (0.53-0.89)	0.96 (0.93-0.99)	0.20 (0.12-0.29)
